# Effects of Nano-Aluminum Nitride on the Performance of an Ultrahigh-Temperature Inorganic Phosphate Adhesive Cured at Room Temperature

**DOI:** 10.3390/ma10111266

**Published:** 2017-11-03

**Authors:** Chengkun Ma, Hailong Chen, Chao Wang, Jifeng Zhang, Hui Qi, Limin Zhou

**Affiliations:** 1 Smart Structures and Advanced Composite Materials Lab, College of Aerospace and Civil Engineering, Harbin Engineering University, Harbin 150001, China; mack@hrbeu.edu.cn (C.M.); jfzhang@hrbeu.edu.cn (J.Z.); qihui@hrbeu.edu.cn (H.Q.); 2 Science and Technology Research Institute, Harbin Engineering University, Harbin 150001, China; 3 Institute of Petrochemistry, Heilongjiang Academy of Science, Harbin 150001, China; 13945092540@163.com; 4 Department of Mechanical Engineering, Hong Kong Polytechnic University, Hong Kong 999077, China; li.min.zhou@polyu.edu.hk

**Keywords:** inorganic phosphate adhesive, modified, ultrahigh-temperature, room temperature curing, shear strength

## Abstract

Based on the optimal proportion of resin and curing agent, an ultrahigh-temperature inorganic phosphate adhesive was prepared with aluminum dihydric phosphate, aluminium oxide (α-Al_2_O_3_), etc. and cured at room temperature (RT). Then, nano-aluminum nitride (nano-AlN), nano-Cupric oxide (nano-CuO), and nano-titanium oxide (nano-TiO_2_) were added into the adhesive. Differential scanning calorimetry was conducted using the inorganic phosphate adhesive to analyze the phosphate reactions during heat treatment, and it was found that 15 wt % nano-AlN could clearly decrease the curing temperature. Scanning electron microscopy was used to observe the microphenomenon of the modified adhesive at ultrahigh-temperature. The differential thermal analysis of the inorganic phosphate adhesive showed that the weight loss was approximately 6.5 wt % when the mass ratio of resin to curing agent was 1:1.5. An X-ray diffraction analysis of the adhesive with 10% nano-AlN showed that the phase structure changed from AlPO_4_(11-0500) to the more stable AlPO_4_(10-0423) structure after heat treatment. The shear strength of the adhesive containing 10% nano-AlN reached 7.3 MPa at RT due to the addition of nano-AlN, which promoted the formation of phosphate and increased the Al^3+^.

## 1. Introduction

With the rapid development of aviation, energy, and metallurgy and the increased usage of large structure in harsh environments, such as high-temperature, high-pressure, and high-speed environments [1,2,3,4,5,6,7], people have increased their demand in the performance of materials, especially high temperature and ultrahigh-temperature adhesive materials. Inorganic phosphate adhesives are high temperature resistant materials based on the development of ceramic, cement, and refractory materials. Due to their character of a high melting point, shrinkage polymerization, and excellent bonding strength, Inorganic phosphate adhesives are highly valued and have potential applications in industry. Moreover, they have become more valuable due to their nontoxic, tasteless, nonpolluting characteristics and especially good high-temperature performance used for bonding metals, ceramics, glass, and other materials [8,9,10,11]. Compared with silicate and organic adhesives, inorganic phosphate adhesives are even able to withstand temperatures of 1300–1700 °C [11,12,13,14]. For large bonding structures, this is obligatory for the adhesive cured at room temperature [15,16,17,18]. Therefore, it is also necessary to develop a kind inorganic phosphate adhesive cured at room temperature.

A lot of research has been carried out on high temperature phosphate materials. The U.S. General Electric Company developed low-cost phosphate high-temperature materials, and these phosphate materials maintained good mechanical and electrical properties at 650 °C when they were cured at 315 °C. A chromium-free phosphate adhesive has been prepared and TG/differential scanning calorimetry (DSC) and XRD methods were used to study the influence of various curing agents on the curing behavior and heat resistance [19]. Phosphate adhesives can convert into ceramics at a temperature of 200 °C, the main constituent of which is AlPO_4_. In addition, various P/Al molar ratios have been used to prepared the adhesives and it was found that the lowest curing temperature occurred when the P/Al ratio was 2.49 [20].

A researcher also studied the curing temperature of adhesives and tried to develop a high temperature adhesive cured at room temperature. A room temperature-cured heat-resistant adhesive for ceramic bonding was manufactured, using low melting point glass (GP) and B_4_C powders as inorganic fillers [21]. The multistage curing mechanisms of phosphate adhesives and the role of hydrogen bonding in curing adhesives were discussed [22,23]. A kind of room temperature-cured iron phosphate ceramic that was expected to be used for treating iron oxide waste was prepared [24]. SiC whisker was used to reinforce the mechanical properties of a phosphate adhesive and it was found that the mechanical performance of the adhesive became enhanced throughout the whole heat-treatment temperature range after adding the appropriate amount (1 wt %) of SiC whisker. The phosphate adhesive could be cured at room temperature and exhibited excellent shear strength; however, the shear strength of the adhesive decreased greatly at high temperature [25]. A phosphate adhesive for bonding C/C composites was manufactured using H_3_PO_4_ and Al(OH)_3_ as the matrix and Si and B_4_C as the inorganic fillers, and it was found that the shear strength decreased when cured at room temperature [26].

Inorganic phosphate adhesives are composed of a base material, curing agent, and filler, and during the process, a few special substances can be added to impart specialized properties. For example, when MgO was added to a curing agent, the curing rate was improved, but the decrease in the crosslink density led to a decrease in the shear strength [27,28]. ZnO has been shown to improve the shear strength of adhesives, but the curing temperature also increases [29]. AlN has strong thermal stability and is alkaline; these properties can promote phosphate reactions. Therefore, to solve the bonding problem of a large scale high temperature resistant structure, we focused on ultrahigh-temperature adhesives (1700 °C) and their properties cured at room temperature, and chose nano-AlN (Guangzhou Jiechuang Trading Co., Ltd., Guangzhou, China) as the curing promoter to modify the adhesive and utilized nano-TiO_2_ (Qingdao Quanhutong Industrial Co., Ltd., Qingdao, China) and nano-CuO (Guangzhou Jiechuang Trading Co., Ltd., Guangzhou, China) to perform a comparative analysis. During our research, a kind of ultrahigh-temperature inorganic phosphate adhesive was developed that could be cured at room temperature. Modified curing agents were used to improve the high-temperature resistance of the adhesive. In addition, the mechanical properties of the adhesive were tested and the modification mechanisms were analyzed.

## 2. Material and Methods

### 2.1. Materials and Manufacturing

**Figure 1:**
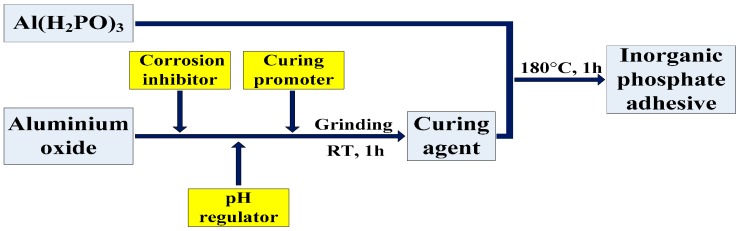
Process for preparing the inorganic phosphate adhesive.

The base material used in the adhesive was aluminum dihydrogen phosphate (Al(H_2_PO_4_)_3_). The preparation of a curing agent is key to the successful development of phosphate system materials. In the process, firstly, the aluminium oxide curing agent acts as the main curing agent, and its particle size and ratio are optimized. The curing agent is mainly composed of aluminium oxide (Al_2_O_3_), and a corrosion inhibitor, pH regulator, curing promoter, etc., are then added, before mixing and grinding the sample thoroughly. Then, the curing agent is blended with the resin and cured at 180 °C for 1 h. The specific process for preparing the adhesive is shown in Figure 1, and the formula of the curing accelerator is listed in Table 1.

### 2.2. Mechanical Tests

Adherents were constituted of silicon carbide ceramics, and the size was 60 × 20 × 3 mm^3^ according to ASTMD1002-05. The adherents were cleaned by tetrahydrofuran and bonded with the adhesive prepared above. After the adhesion process, the test specimens were placed in a fixture within an oven. The overlapped length was 10 mm.

**Figure 2:**
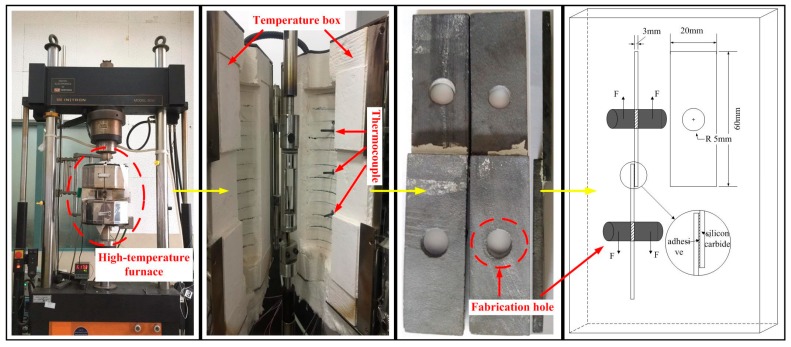
Schematic diagram of a single lap joint and the high-temperature tension tester.

The mechanical tests were performed at 25, 1000, 1200, 1500, and 1700 °C using an INSTRON 8032 high-temperature tensile machine (INSTRON, Shanghai, China). In order to identify the low temperature performance of this adhesive, we also tested its shear strength at temperatures of −120 °C and −55 °C, cooling it with liquid nitrogen. The performances at 25 °C and 1000 °C were tested online, and the shear strength (1200, 1500, and 1700 °C) was tested after heating treatment. The INSTRON 8032 is equipped with a high-temperature furnace, data acquisition system, and thermocouple, as shown in Figure 2. Five specimens of each group were tested, and the average values were determined. In this experiment, the loading velocity of the stretching machine was 2 mm/min. The loading process was halted once the tensile meter dropped to 20%.

### 2.3. Differential Thermal Analysis Tests

Differential thermal analysis (DTA, 200PC, NETZSCH, Selb, Germany) was used to analyze the effects of the curing agent content and curing method on the curing temperature and degree of reactivity. During the process, the heating rate was 10 °C/min in an argon environment with a mass flow of 30 mL/min from RT to 900 °C.

### 2.4. X-ray Diffraction Tests

The specimens were heated to 800, 1000, and 1200 °C, respectively, for 10 min with a heating rate of 50 °C/h in a muffle furnace. Then, X-ray diffraction (Y-500, Dandong, China) with CuKα radiation was used to study the effects of the curing agent on the crystal structure of the adhesive from 10° to 80°, and the scanning rate was 0.1°/S.

### 2.5. Scanning Electron Microscope Tests

SEM (JEM1200EX, NEC Corporation, Tokyo, Japan) was used to observe the microtopography while varying the curing agent content and the temperature.

## 3. Results and Discussion

### 3.1. Thermal Properties

**Figure 3:**
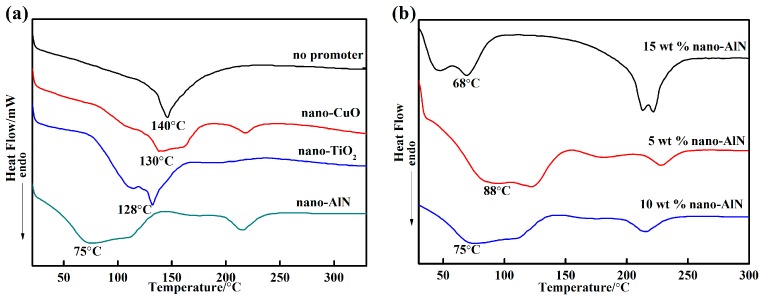
DSC curves of the various adhesives: (**a**) DSC curves of the adhesives with various promoters; and (**b**) DSC curves of the adhesives with various proportions of nano-AlN.

DSC curves of the adhesives with various curing promoters are shown in Figure 3a. The endothermic peaks of the adhesives without a promoter and with nano-CuO, nano-TiO_2_, and nano-AlN were at 140 °C, 130 °C, 128 °C, and 75 °C, respectively, and they all noticeably decreased. Notably, the curing temperature decreased after adding the curing promoter, and the addition of nano-AlN resulted in the most significant reduction in the curing temperature. A significant reduction was observed because the aluminium dihydrogen phosphate is acidic, and the basicity of nano-AlN is stronger than that of nano-TiO_2_ and nano-CuO. Hence, adding nano-AlN leads to strong acid-base reactions and promotes the formation of phosphate. Figure 3b shows DSC curves of the adhesives with various proportions of nano-AlN. From the figure, the curing temperature with 15% nano-AlN was the minimum, and the endothermic peak occurred at 68 °C. The endothermic peak of 5% nano-AlN occurred at 88 °C. Nano-AlN and AlPO_4_ underwent an acid-base reaction; therefore, the 15% nano-AlN clearly promoted the reaction and thus reduced the curing temperature. The endothermic peak of the adhesive with 5 wt % nano-AlN at 88 °C is greater than that after adding 10% nano-AlN, as shown in Figure 3b; this result indicates that the 5 wt % dosage of the curing agent was too small and could not promote the reaction.

**Figure 4:**
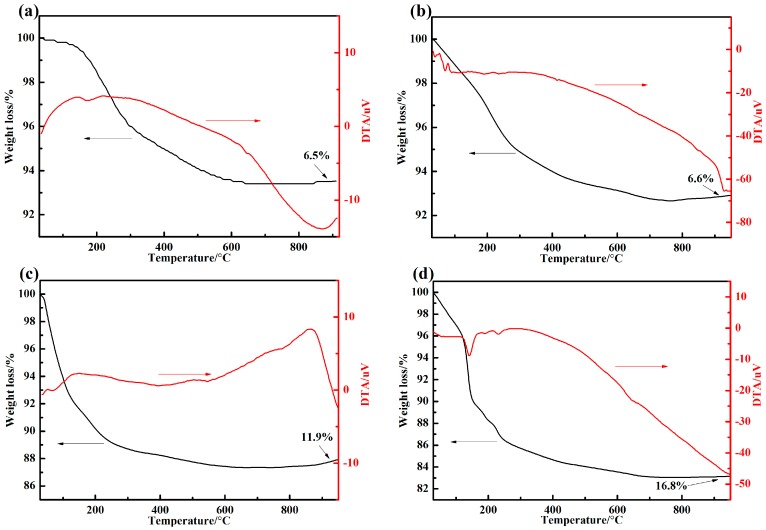
TG/DTA results of the adhesives with various proportions of resin and curing agent: (**a**) resin: curing agent = 1:1.5; (**b**) resin: curing agent = 1:1; (**c**) resin: curing agent = 1:0.5; and (**d**) resin: curing agent = 1:0.4.

TG curves of the adhesive are shown as in Figure 4 for various proportions of resin and curing agent. When the temperature increased, the TG curve decreased. This phenomenon could be explained as the aluminium dihydrogen phosphate being decomposed into water and aluminum phosphate at high temperature, and the volatilization of water caused weight loss. The lower the curing agent content, the more unreacted aluminium dihydrogen phosphate. Excessive moisture volatilization led to severe weight loss. From the figures, it is found that the adhesive heat resistance was enhanced as the curing agent content increased. But it also leads to the increase of viscosity and a poor manufacturing process. When the content ratio of resin and curing agent content was more than 1:1, the TG curves reduced slowly, which indicated that its heat resistance decreased. From Figure 4a,b, it can be found that the weight loss rate was 6.5% and 6.6% with resin and curing agent at a mass ratio of 1:1.5 and 1:1, respectively. When the content ratio of the resin to curing agent was 1 to 1.5, the weight loss rate of the adhesive was 6.5%. The adhesive weight loss rate reached 16.8% in Figure 4d when the content of the curing agent was 40 wt %.

### 3.2. Shear Strength

**Figure 5:**
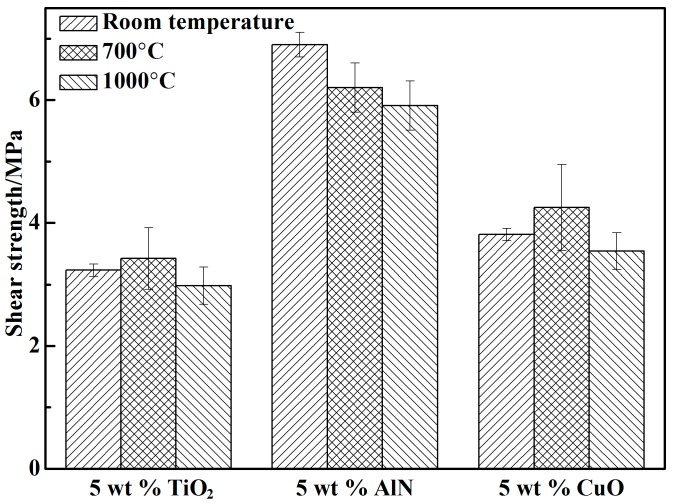
The shear strengths of the adhesives with various curing agents.

The shear strengths of the adhesives with various curing promoters are shown in Figure 5. From Figure 5, the shear strength of the adhesive with 5 wt % nano-AlN was higher than that of the adhesives with nano-TiO_2_ or nano-CuO because the addition of nano-AlN increased the amount of Al^3+^ in the adhesive. The shear strength was 6.9 MPa and 6.2 MPa at RT and 700 °C, respectively. When the temperature increased to 1000 °C, the shear strength decreased to 5.9 MPa due to phosphate pyrolysis.

**Figure 6:**
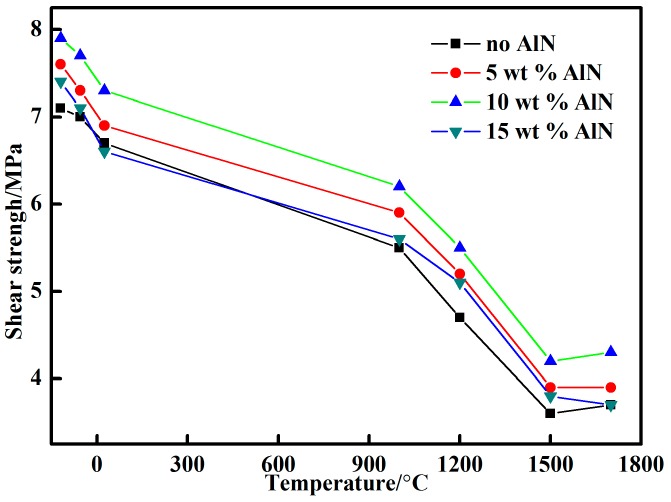
The shear strengths of the adhesives with various curing agents at different temperatures.

From Figure 6, the shear strength of the 10 wt % nano-AlN adhesive was higher than that of the adhesives with other proportions of nano-AlN. It could also be found that the shear strength increased at low temperature compared with other temperatures. This phenomenon could be explained as the low temperature postponing the movement of molecular chains in phosphate polymers, leading to strength reinforcement of the adhesive. The shear strength of the 10 wt % nano-AlN joints decreased from 7.3 MPa at RT to 6.3 MPa at 1000 °C and to approximately 5.5 MPa at 1200 °C. When the heat-treatment temperature increased from 1500 °C to 1700 °C, the 10 wt % nano-AlN increased from 4.2 MPa to 4.3 MPa. From the integrated analysis, we determined that the nano-AlN was easily hydrolyzed and adding nano-AlN enhanced the formation of phosphate. Moreover, the addition of nano-AlN increased the content of Al^3+^ in the adhesive and increased the strength. However, when adding excess nano-AlN, the reaction resulted in the formation of a large number of NH_3_ groups, which led to a higher adhesive porosity and affected the strength. When the temperature exceeded 1500 °C, the shear strength increased because the main component of the adhesive was Al (H_2_PO_4_)_3_. When heated to a certain temperature, aluminium dihydrogen phosphate became aluminum pyrophosphate and aluminum metaphosphate and polymerization occurred. When the temperature exceeded 1500 °C, the aluminum metaphosphate polymer decomposed, forming aluminum phosphate (AlPO_4_) and phosphorus pentoxide (P_2_O_5_). In addition, P_2_O_5_ also generated AlPO_4_ in the presence of Al_2_O_3_ to strengthen the bonding strength. Therefore, we concluded that the promoter greatly influenced the shear strength of the adhesive after curing at RT.

### 3.3. X-ray Diffraction Analysis

**Figure 7:**
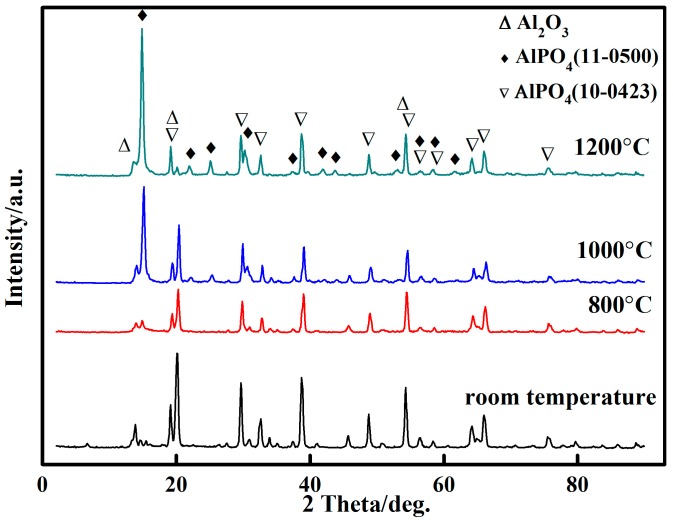
XRD spectra of the adhesive at various temperatures.

The adhesive specimens were heat treated at temperatures of 800, 1000, and 1200 °C, and the XRD spectra of the specimens are illustrated in Figure 7. The results show that the diffraction peak of phosphate (AlPO_4_(11-0500)) drastically increased and that of aluminium oxide (α-Al_2_O_3_) decreased as the treatment temperature increased. The diffraction peak of phosphate (AlPO_4_(11-0500)) was unusually sharp at 1200 °C; this result indicated that some of the phosphate (AlPO_4_(10-0423)) transformed into a more stable phosphate crystal (AlPO_4_(11-0500)).

### 3.4. Micromorphology

**Figure 8:**
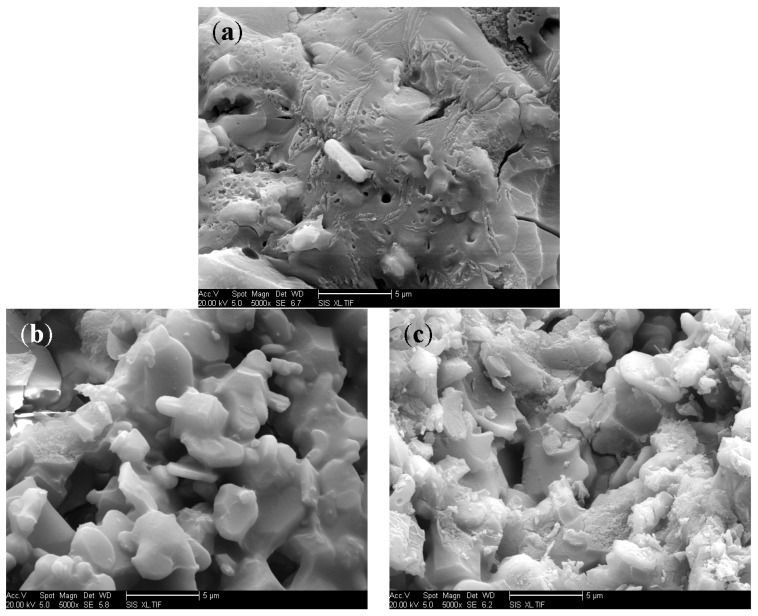
Micrographs of the interface of the adhesive: (**a**) 5 wt % nano-AlN; (**b**) 10 wt % nano-AlN; and (**c**) 15 wt % nano-AlN.

Figure 8 shows the effect of the promoter content on the morphology of the bonding layer. The numbers of pores, cracks, and particles in Figure 8a of the adhesive with 5 wt % nano-AlN were significantly fewer than those of the 10 wt % nano-AlN and 15 wt % nano-AlN adhesives, indicating that adding 5 wt % nano-AlN improved the integrity of the bonding layer. Because nano-AlN is alkaline, adding excess nano-AlN intensifies the reaction between nano-AlN and phosphoric acid, resulting in a large number of NH_3_ groups. The volatilization of NH_3_ causes the surface porosity of the adhesive to increase and reduces the adhesive strength. Surely, a cohesive surface without or with a minimum number of pores and cracks would enhance the shear strength [30]. However, decreasing the amount of nano-AlN did not promote the phosphate reaction, resulting in a higher curing temperature.

(1) $$\mathrm{AlN}+H_{3}{\mathrm{PO}}_{4}={\mathrm{AlPO}}_{4}+{\mathrm{NH}}_{3}$$

**Figure 9:**
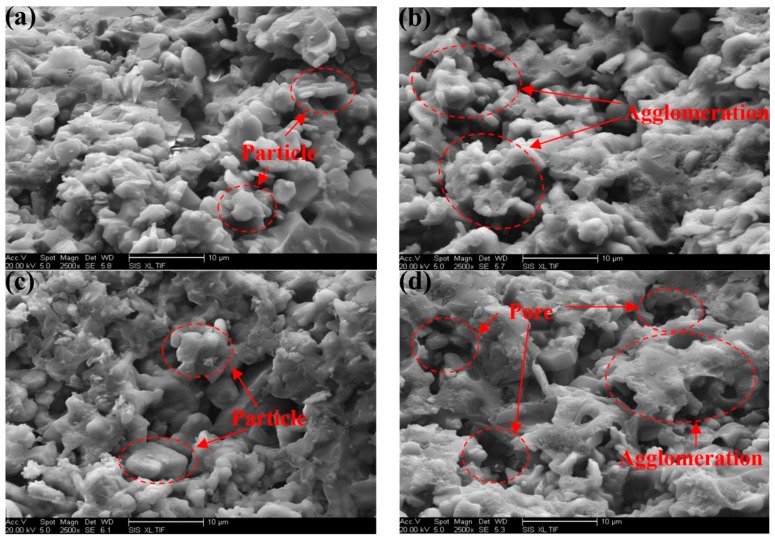
Micrographs of the surface of the adhesive at different temperature: (**a**) 1000 °C; (**b**) 1500 °C; (**c**) 1700 °C; and (**d**) 2000 °C.

Figure 9 shows micrographs of the adhesive surfaces modified with the curing agents after being heated at various temperatures. In the figure, many rough particles can be observed on the surfaces, which explains why the shear strengths obtained after heating were lower than those at a normal temperature. With the increase in temperature, agglomeration was observed in Figure 9b. When the temperature increased to 1700 °C, the sizes of the pores decreased, and slight crosslinking curing appeared, as shown in Figure 9c. This phenomenon can be explained because Al_2_O_3_ further reacted with the base material with the increasing temperature. When the temperature increased to 2000 °C, a small amount of the substance decomposed, as shown in Figure 9d, resulting in a significant increase in porosity and agglomeration and decreasing the shear strength.

## 4. Conclusions

This study addressed the performance of an ultrahigh-temperature inorganic phosphate adhesive that was cured at RT. The following conclusions were drawn:

1. An ultrahigh-temperature inorganic phosphate adhesive cured at RT was prepared with aluminium dihydrogen phosphate as the resin and aluminium oxide (α-Al_2_O_3_), etc., as the curing agent. When the content of the curing agent was more than that of resin, it had less effect on the thermal stability of the adhesive. The inorganic phosphate adhesive could be prepared with a ratio of 1:1 in consideration of heat resistance and the manufacturing process.
2. The inorganic phosphate adhesive has excellent heat resistance. Addtionally, the promoters and curing agents significantly affected the curing temperature. The phase structure of the adhesive with 10 wt % nano-AlN changed from AlPO_4_(11-0500) to the more stable AlPO_4_(10-0423) at 1200 °C.
3. A proper amount of nano-AlN clearly improved the shear strength of the adhesive because of the increased Al^3+^ content. The shear strength of the adhesive with 10 wt % nano-AlN was higher than that of the adhesives with other proportions of nano-AlN. A spot of nano-AlN did not fully promote the reaction between the resin and the curing agent. Excess nano-AlN instead decreased the shear strength due to the increase of porosity.

## Figures and Tables

**Table 1:** Curing promoter formulations.

Formulation	Curing Promoter	Dosage (wt %)	Particle Size (μm)	Purity
a	/			
b	Nano-AlN	15	1	99.5%
c	Nano-AlN	10	1	99.5%
d	Nano-AlN	5	1	99.5%
e	Nano-TiO_2_	5	1	99.5%
f	Nano-CuO	5	1	99.5%

## References

[1] Dadras P., Mehrotra G.M. (1994). Joining of carbon-carbon composites by graphite formation. J. Am. Ceram. Soc..

[2] Dadras P., Mehrotra G.M. (1993). Solid state diffusion bonding of carbon-carbon composites with borides and carbides. J. Am. Ceram. Soc..

[3] Wang M., Hu X., Xu X., Yun Z., Liu J., Du H., Guo A. (2015). A user-friendly heat-resistant modified polymer-based adhesive for joining and repair of carbon/carbon composites. Mater. Des..

[4] Marques E.A.S., da Silva L.F.M., Flaviani M. (2015). Testing and simulation of mixed adhesive joints for aerospace applications. Compos. B Eng..

[5] Katsiropoulos C.V., Chamos A.N., Tserpes K.I., Pantelakis S.G. (2012). Fracture toughness and shear behavior of composite bonded joints based on a novel aerospace adhesive. Compos. B Eng..

[6] Kadiyala A.K., Sharma M., Bijwe J. (2016). Exploration of thermoplastic polyimide as high temperature adhesive and understanding the interfacial chemistry using XPS, ToF-SIMS and Raman spectroscopy. Mater. Des..

[7] Yang Z., Zhang X., Liu X., Guan X., Zhang C., Niu Y. (2017). Polyglycerol-based organic-inorganic hybrid adhesive with high early strength. Mater. Des..

[8] Han H.J., Kim D.P. (2003). Studies on curing chemistry of aluminum-chromium-phosphates as low temperature curable binders. J. Sol-Gel Sci. Technol..

[9] Wang C., Huang Y.D., Wang B. (2006). Study on heat-resistant property of adhesive/carbon-carbon composites joints. Int. J. Adhes. Adhes..

[10] Geng H., Liu J., Guo A., Ren S., Xu X., Liu S. (2016). Fabrication of heat-resistant syntactic foams through binding hollow glass microspheres with phosphate adhesive. Mater. Des..

[11] Wang M., Liu J., Du H., Hou F., Guo A., Zhao Y., Zhang J. (2014). A new practical inorganic phosphate adhesive applied under both air and argon atmosphere. J. Alloy. Compd..

[12] Srivastava V.K. (2011). Effect of carbon nanotubes on the strength of adhesive lap joints of C/C and C/C-SiC ceramic fibre composites. Int. J. Adhes. Adhes..

[13] Zhang Y., Luo R., Zhang J., Xiang Q. (2011). The reinforcing mechanism of carbon fiber in composite adhesive for bonding carbon/carbon composites. J. Mater. Process. Technol..

[14] Liu Z.X., Sun R.N., Mao Z.P., Wang P.C. (2012). Effects of phosphate pretreatment and hot-humid environmental exposure on static strength of adhesive-bonded magnesium AZ31 sheets. Surf. Coat. Technol..

[15] Levine S.R., Opila E.J., Halbig M.C., Kiser J.D., Singh M., Salem J.A. (2002). Evaluation of ultra-high temperature ceramics for aeropropulsion use. J. Eur. Ceram. Soc..

[16] Fan R.L., Zhang Y., Li F., Zhang Y.X., Sun K., Fan Y.Z. (2001). Effect of high-temperature curing on the crosslink structures and dynamic mechanical properties of gum and N330-filled natural rubber vulcanizates. Polym. Test..

[17] Lin Y.J., Tu S.H. (2009). Joining of mullite ceramics with yttrium aluminosilicate glass interlayers. Ceram. Int..

[18] Rosa R., Veronesi P., Han S., Casalegno V., Salvo M., Colombini E., Leonelli C., Ferraris M. (2013). Microwave assisted combustion synthesis in the system Ti-Si-C for the joining of SiC: Experimental and numerical simulation results. J. Eur. Ceram. Soc..

[19] Hong L.Y., Han H.J., Ha H., Lee J.Y., Kim D.P. (2007). Development of Cr-free aluminum phosphate binders and their composite applications. Compos. Sci. Technol..

[20] Fernando J.A., Chung D.D.L. (2001). Improving an alumina fiber filter membrane for hot gas filtration using an acid phosphate binder. J. Mater. Sci..

[21] Zhang J., Luo R., Jiang M., Xiang Q., Li J. (2011). The preparation and performance of a novel room-temperature-cured heat-resistant adhesive for ceramic bonding. Mater. Sci. Eng. A.

[22] Karpukhin I.A., Vladimirov V.S., Moizis S.E. (2005). A Mechanism for Phosphate Hardening and Prospects for the Use of Metal Phosphate Materials (A Review). Part I. The Nature of Hydrogen Bonding and Its Function in the Mechanism of Phosphate Hardening. Refract. Ind. Ceram..

[23] Karpukhin I.A., Vladimirov V.S., Moizis S.E. (2005). A Mechanism for Phosphate Hardening and Prospects for the Use of Metal Phosphate Materials (An Overview). Part II. Adhesive Properties of Binding Phosphate Materials. Refract. Ind. Ceram..

[24] Wagh A.S., Jeong S.Y. (2003). Chemically bonded phosphate ceramics: III, reduction mechanism and its application to iron phosphate ceramics. J. Am. Ceram. Soc..

[25] Wang M., Liu J., Du H., Guo A., Tao X., Dong X., Geng H. (2015). A SiC whisker reinforced high-temperature resistant phosphate adhesive for bonding carbon/carbon composites. J. Alloys Compd..

[26] Wang M., Liu J., Du H., Hou F., Guo A., Liu S., Dong X. (2014). Joining of C/C composites by using B_4_C reinforced phosphate adhesive. Ceram. Int..

[27] Shah S., Shweta Sharma A., Gupta M.N. (2004). Biodiesel Preparation by Lipase-Catalyzed Transesterification of Jatropha Oil. Energy Fuels.

[28] Srivastava A., Prasad R. (2000). Triglycerides-based diesel fuels. Renew. Sustain. Energy Rev..

[29] Xiaobo Y., Yudong H., Jie Z., Hailin C. (2005). Preparation and Properties of Phosphate Base Heat-resisting Composites. Chem. Adhes..

[30] Liu S., Cheng X., Zhang Q., Zhang J., Bao J., Guo X. (2016). An investigation of hygrothermal effects on adhesive materials and double lap shear joints of CFRP composite laminates. Compos. B Eng..

